# Aloe emodin isolated from *Cassiae* semen upregulates IFITM1 to suppress hepatitis B virus replication via the interferon signaling pathway

**DOI:** 10.1128/msphere.00312-26

**Published:** 2026-06-11

**Authors:** Xin Wen, Xincan Yu, Yuxin Hu, Jiamin Chen, Dongwei Zhang, Yalin Hou, Shui Liu, Xuemeng Shi, Jun Xu

**Affiliations:** 1 College of Life Sciences, Zhengdong New District Longzi Lake Campus, Henan Agricultural University 70573, Zhengzhou, Henan, China; 2 Henan Province Engineering Research Center of Crop Synthetic Biology, College of Life Sciences, Henan Agricultural University 70573, Zhengzhou, China; Dartmouth College Geisel School of Medicine, Hanover, New Hampshire, USA

**Keywords:** *Cassiae *semen, aloe emodin, hepatitis B virus, interferons, IFITM

## Abstract

The seeds of *Cassia obtusifolia* L. or *Cassia tora* L., known as *Cassiae* semen (juemingzi in Chinese), has been traditionally utilized for its hepatoprotective, lipid-lowering, anti-inflammatory, and immunomodulatory properties. However, their effects on hepatitis B virus (HBV), which causes chronic infection leading to liver cirrhosis and hepatocellular carcinoma, remain largely unexplored. In this study, we prepared *Cassiae* semen water extract (CWE) and evaluated its impact on HBV replication using HepAD38 cells. Our results showed that CWE suppressed HBV replication in a concentration-dependent manner. To identify the bioactive components, several compounds derived from *Cassiae* semen, including cassiaside C, rubrofusarin-6-O-β-gentiobioside, aurantioobtusin, and aloe emodin, were tested for their anti-HBV activity. Among them, aloe emodin exhibited the most potent anti-HBV activity. Further mechanistic investigations indicated that the antiviral effects of CWE and aloe emodin were associated with the interferon (IFN) signaling pathway. Treatment with both CWE and aloe emodin markedly upregulated the expression of type I IFN and the IFN-stimulated gene (ISG) IFITM1. Knockout of IFITM1 expression significantly weakened the anti-HBV activity of CWE and aloe emodin, demonstrating that the IFN-IFITM1 axis serves as a key pathway mediating the inhibitory effects of *Cassiae* semen on HBV replication. Collectively, our findings demonstrate for the first time that *Cassiae* semen extract and its bioactive constituent aloe emodin exert potent anti-HBV efficacy by modulating the interferon signaling pathway, thereby highlighting their potential as novel therapeutic agents for the treatment of chronic hepatitis B.

**IMPORTANCE:**

CHB caused by HBV is a major global health threat, often progressing to liver cirrhosis and hepatocellular carcinoma, with limited effective therapies. *Cassiae* semen, a widely used food-medicine homology herb in traditional Chinese medicine, is known for hepatoprotective properties, but its anti-HBV potential remains unexploited. This study identifies aloe emodin as the key bioactive component of *Cassiae* semen water extract that suppresses HBV replication via activating the host interferon-IFITM1 pathway with low cytotoxicity. These findings not only uncover a novel natural anti-HBV agent but also bridge traditional herbal medicine with modern antiviral drug development, offering a promising, safe candidate for HBV-associated disease treatment.

## INTRODUCTION

Chronic infection with hepatitis B virus (HBV), a 3.2 kb DNA virus, represents a major global health burden that can progress to severe liver diseases, including liver cirrhosis and hepatocellular carcinoma (HCC) (1, 2). The host interferon (IFN) response serves as a pivotal defense mechanism against HBV infection, and type I IFN has been approved as a therapeutic agent for chronic hepatitis B (CHB). However, its clinical efficacy is constrained by inflammatory adverse effects associated with low antiviral efficacy and cure rate (3, 4). Accumulating interferon-stimulated genes (ISGs) have been reported to exert anti-HBV activity by impeding HBV DNA replication and RNA transcription, as well as reducing hepatitis B e antigen secretion (5). Our recent studies further demonstrated that the anti-HBV activity of ISG member-interferon-induced transmembrane protein 1 (IFITM1) was tightly regulated by post-translational modifications (PTMs) such as S-palmitoylation (6, 7). However, the therapeutic strategies targeting IFITM1 for chronic hepatitis B, as well as the specific molecular mechanisms underlying IFITM1-mediated suppression of HBV replication, remain largely unexplored.

*Cassiae* semen (juemingzi in Chinese), the dried seed of Fabaceae plants *Cassia obtusifolia* L. or *Cassia tora* L., is a well-documented traditional Chinese medicinal herb with notable hepatoprotective, anti-hepatic steatosis, anti-inflammatory, and immunomodulatory properties (8, 9). Extract derived from *Cassiae* semen is abundant in bioactive components, with anthraquinones and naphthopyranones as its major pharmacologically active substances. Anthraquinones represent the predominant bioactive constituents of *Cassiae* semen, which exhibit significant anti-inflammatory and antioxidant activities, mainly including aloe emodin, emodin, and aurantioobtusin. Naphthopyranones exert primary effects in hepatoprotection and generally exist in the form of glycosides, with representative components including cassiaside C and rubrofusarin-6-O-β-gentiobioside, etc (8, 10, 11). The bioactive components of *Cassiae* semen are commonly extracted by water or ethanol percolation. Notably, the aqueous extract shows relatively low toxicity and is rich in bioactive components, including Aloe emodin, aurantioobtusin, cassiaside C, and rubrofusarin-6-O-β-gentiobioside (12–14). *Cassiae* semen has been currently utilized in China as a food-medicine homology ingredient for the adjuvant therapy of fatty liver, diabetes complications, and other metabolic diseases (8); nevertheless, its specific pharmacological molecular mechanisms and potential applications in CHB treatment remain largely elusive.

Specifically regarding the regulatory role of *Cassiae* semen in the process of pathogen infection, mounting evidence has shown that it exerts inhibitory effects on infections caused by influenza A virus (H1N1 and H3N2 subtypes), influenza B virus (15), SARS-CoV (16), SARS-CoV-2 (17), and *Staphylococcus aureus* (8, 18). Although several food-medicine homology substances and traditional Chinese nedicine (TCM), such as green tea and *Scutellaria barbata* D. Don, exhibit anti-HBV effects by suppressing viral replication (19–21), the effects of hepatoprotective *Cassiae* semen on HBV replication remain uncharacterized. This critical knowledge gap presents a compelling rationale for investigating its potential as a new therapeutic agent against HBV.

Herein, we systematically evaluated the anti-HBV activity of the *Cassiae* semen water extract (CWE) and identified its key bioactive constituents. Several major pharmacologically active anthraquinones and naphthopyrones were screened for antiviral potency, among which aloe emodin exhibited robust anti-HBV activity even at low concentrations and with short-term treatment. Mechanistically, CWE or aloe emodin potently activated the type I interferon signaling pathway, with the most prominent upregulation of IFITM1 observed among ISGs with anti-HBV properties. Notably, IFITM1 knockout attenuated CWE/aloe emodin-mediated anti-HBV activity, indicating that CWE or aloe emodin exerts anti-HBV effects primarily via the IFN-IFITM1 signaling axis. Collectively, this study establishes a theoretical foundation for the clinical application of *Cassiae* semen in chronic hepatitis B therapy and identifies aloe emodin as a promising natural product candidate with potent anti-HBV activity.

## RESULTS

### Aqueous extract of *Cassiae* semen suppresses HBV replication

**Fig 1:**
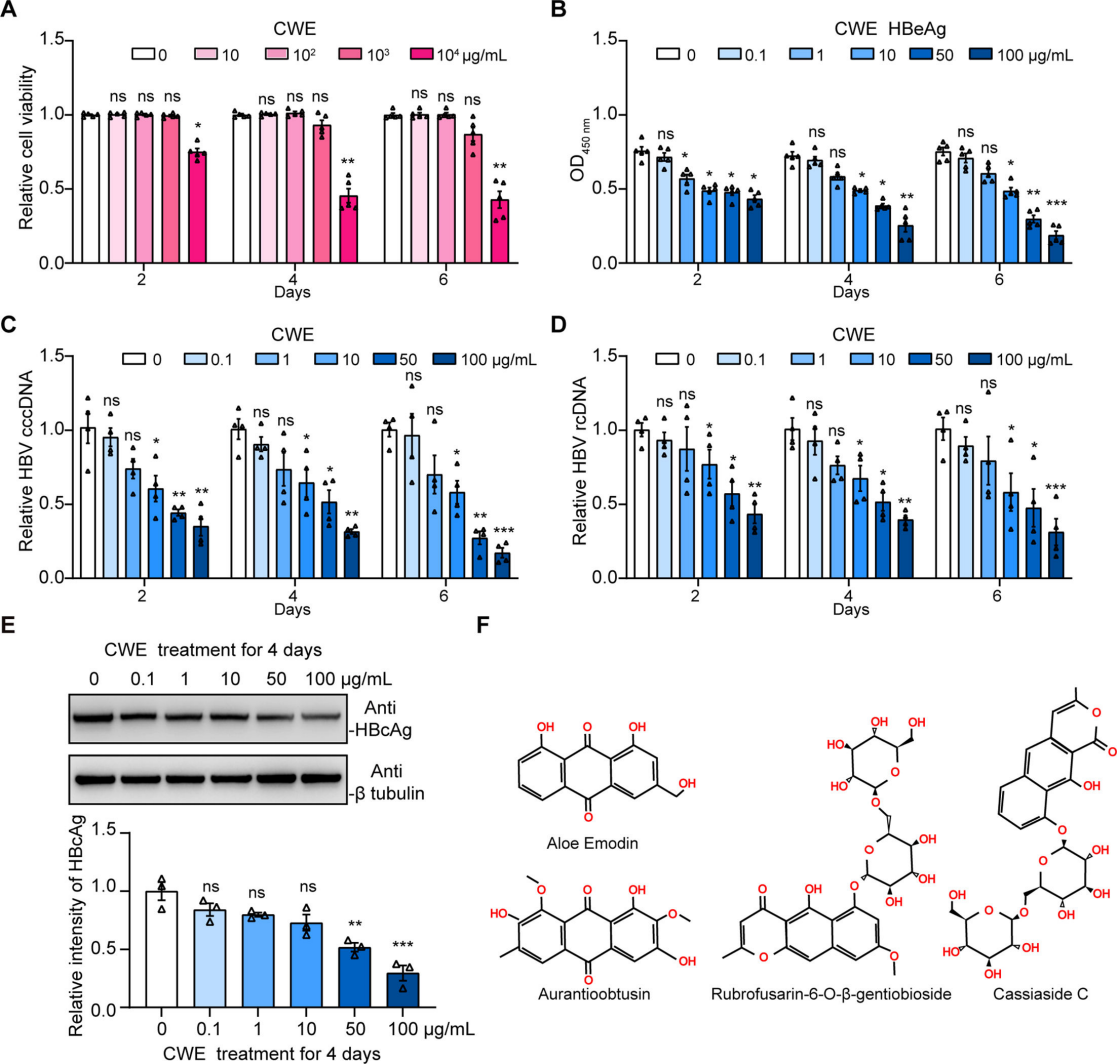
CWE treatment inhibits the replication of HBV. (**A**) Cell viability of HepAD38 cells treated with CWE at concentrations of 0, 10, 100, 1,000, and 10,000 μg/mL for 2, 4, and 6 days. (**B**) ELISA analysis of HBeAg levels in the culture medium of HepAD38 cells treated with CWE (0, 0.1, 1, 10, 50, and 100 μg/mL) for 2, 4, and 6 days. (**C and D**) The levels of HBV cccDNA (**C**) and rcDNA (**D**) in HepAD38 cells treated with CWE (0, 0.1, 1, 10, 50, and 100 μg/mL) for 2, 4, and 6 days. (**E**) Western blotting analysis of HBcAg amounts in HepAD38 cells treated with CWE (0, 0.1, 1, 10, 50, and 100 μg/mL) for 4 days. The relative intensities of HBcAg bands in each group were quantified using ImageJ software. (**F**) Chemical structures of aloe emodin, aurantioobtusin, rubrofusarin-6-O-β-gentiobioside, and cassiaside C. Data are presented as mean ± SEM from three independent experiments. ns, no significant difference; *P < 0.05; **P < 0.01; ***P < 0.001 (one-way ANOVA).

As a TCM herb, *Cassiae* semen has long been recognized for its hepatoprotective effects (8); however, its potential antiviral properties, particularly against the infection of the hepatitis B virus, remain largely unclear. To systematically evaluate its anti-HBV activity, the *Cassiae* semen water extract (CWE) was prepared and lyophilized into a stable powder form. We treated HepAD38 cells, a tetracycline-inducible HBV replicating cell line, with CWE at a broad concentration range (0, 10, 100, 1,000, and 10,000 μg/mL) for 2, 4, and 6 days. Cell viability assessment using the CCK-8 assay revealed that only the highest concentration (10,000 μg/mL) induced detectable cytotoxicity, indicating a favorable safety profile for CWE at clinically relevant doses (Fig. 1A).

To quantitatively evaluate the antiviral efficacy of CWE against HBV, multiple hallmark indices of HBV replication were measured following treatment with a serial concentration gradient of CWE (0, 0.1, 1, 10, 50, and 100 μg/mL). We measured the level of hepatitis B e antigen (HBeAg), which is a serological marker indicative of active HBV replication and high viral infectivity, in the cell culture supernatant via ELISA. Meanwhile, intracellular covalently closed circular DNA (cccDNA, the stable nuclear replication template that drives persistent HBV infection) and relaxed circular DNA (rcDNA, the genomic form of HBV in virions that functions as a precursor for cccDNA synthesis) were quantified using qRT-PCR (Fig. 1B through D). The results revealed a robust, concentration-dependent reduction in all three viral parameters throughout the treatment course. This CWE-mediated suppression of HBV replication was further validated by western blotting analysis, which demonstrated a concomitant reduction in the expression of hepatitis B core antigen (HBcAg), a core structural component of HBV that marks active viral replication within hepatocytes, in CWE-treated HepAD38 cells (Fig. 1E).

Given the complex composition of *Cassiae* semen, we sought to identify the specific bioactive constituents responsible for the anti-HBV activity. Based on phytochemical profiling, the major pharmacologically active compounds in *Cassiae* semen are known to be anthraquinones and naphthopyranones (22). From these categories, we selected two representative anthraquinones—aurantioobtusin and aloe emodin—and two naphthopyranones—cassiaside C and rubrofusarin-6-O-β-gentiobioside—all of which are detectable in the aqueous extract of *Cassiae* semen (12–14), for further investigation (Fig. 1F). Subsequent experiments were designed to systematically evaluate the individual contributions of these purified compounds to the anti-HBV activity observed with the complete extract.

### Effects of distinct *Cassiae* semen bioactive constituents on HBV replication

**Fig 2:**
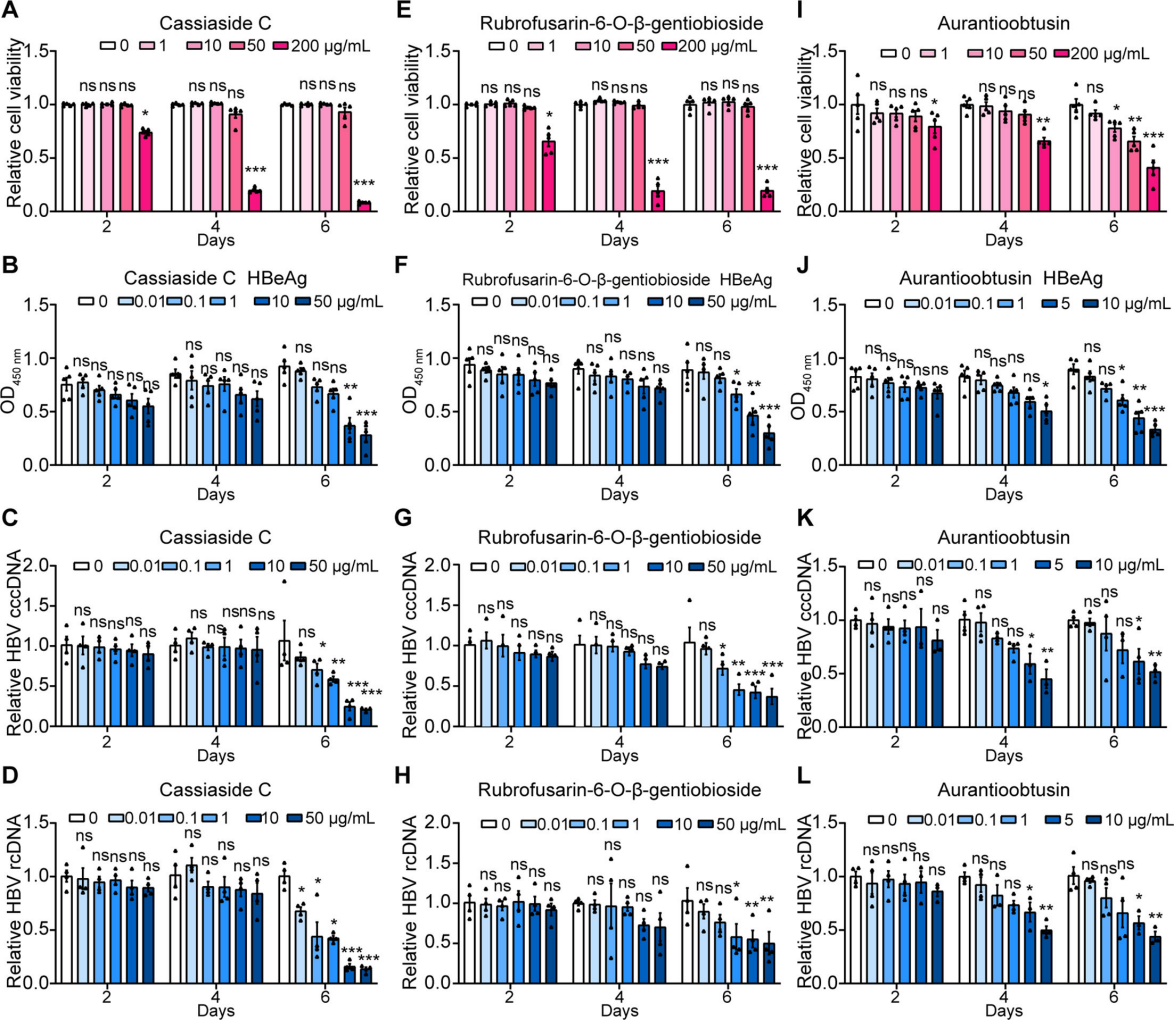
The effects of cassiaside C, rubrofusarin-6-O-β-gentiobioside, and aurantioobtusin on HBV replication. (**A**) CCK-8 assay to evaluate the viability of HepAD38 cells treated with cassiaside C (0, 1, 10, 50, and 200 μg/mL) for 2, 4, and 6 days. (**B**) ELISA analysis of HBeAg levels in the culture supernatant of HepAD38 cells treated with cassiaside C (0, 0.01, 0.1, 1, 10, and 50 μg/mL) for 2, 4, and 6 days. (**C and D**) The levels of HBV cccDNA (**C**) and rcDNA (**D**) in HepAD38 cells treated with cassiaside C (0, 0.01, 0.1, 1, 10, and 50 μg/mL) for 2, 4, and 6 days. (**E**) CCK-8 assay to assess the viability of HepAD38 cells treated with rubrofusarin-6-O-β-gentiobioside (0, 1, 10, 50, and 200 μg/mL) for 2, 4, and 6 days. (**F**) ELISA analysis of HBeAg levels in the culture medium of HepAD38 cells treated with rubrofusarin-6-O-β-gentiobioside (0, 0.01, 0.1, 1, 10, and 50 μg/mL) for 2, 4, and 6 days. (**G and H**) The levels of HBV cccDNA (**G**) and rcDNA (**H**) in HepAD38 cells treated with rubrofusarin-6-O-β-gentiobioside (0, 0.01, 0.1, 1, 10, and 50 μg/mL) for 2, 4, and 6 days. (**I**) CCK-8 assay to determine the viability of HepAD38 cells treated with aurantioobtusin (0, 1, 10, 50, and 200 μg/mL) for 2, 4, and 6 days. (**J**) ELISA analysis of HBeAg levels in the culture medium of HepAD38 cells treated with aurantioobtusin (0, 0.01, 0.1, 1, 5, and 10 μg/mL) for 2, 4, and 6 days. (**K and L**) The levels of HBV cccDNA (**K**) and rcDNA (**L**) in HepAD38 cells treated with aurantioobtusin (0, 0.01, 0.1, 1, 5, and 10 μg/mL) for 2, 4, and 6 days. Data are presented as mean ± SEM from three independent experiments. ns, no significant difference; *P < 0.05; **P < 0.01; ***P < 0.001 (one-way ANOVA).

Based on the initial screening of candidate compounds, we first systematically evaluated the cytotoxic effects of naphthopyranones (cassiaside C and rubrofusarin-6-O-β-gentiobioside) on HepAD38 cells. Cells were treated with gradient concentrations (0, 1, 10, 50, and 200 μg/mL) of cassiaside C or rubrofusarin-6-O-β-gentiobioside for 2, 4, and 6 days, respectively. The CCK-8 assay results demonstrated that exposure to 200 μg/mL of either cassiaside C or rubrofusarin-6-O-β-gentiobioside significantly compromised cell viability compared to the untreated control group (Fig. 2A and E). Guided by these cytotoxicity profiles, we subsequently utilized a refined concentration range (0, 0.01, 0.1, 1, 10, and 50 μg/mL) to treat HepAD38 cells for the same duration to assess antiviral efficacy. Measurement of HBeAg secretion into culture supernatant (Fig. 2B and F), along with quantification of intracellular cccDNA (Fig. 2C and G) and rcDNA (Fig. 2D and H), indicated that neither 2-day nor 4-day treatment with cassiaside C or rubrofusarin-6-O-β-gentiobioside produced significant suppression of viral replication. However, extending the treatment duration to 6 days resulted in a modest yet concentration-dependent inhibitory effect on multiple key parameters of the HBV life cycle.

We next investigated the cytotoxicity and antiviral potential of anthraquinone compounds. Treatment of HepAD38 cells with Aurantioobtusin (0, 1, 10, 50, and 200 μg/mL) for 2, 4, and 6 days revealed substantial cytotoxicity. Notably, 200 μg/mL aurantioobtusin treatment for just 2 days, or exposure to 10 μg/mL for 6 days, already significantly reduced cell viability (Fig. 2I). This cytotoxic effect exhibited both concentration and time dependence, suggesting limited therapeutic applicability. Corresponding evaluations in HepAD38 cells showed that 2-day aurantioobtusin treatment had no measurable impact on HBeAg production (Fig. 2J), cccDNA (Fig. 2K), or rcDNA levels (Fig. 2L). While extended treatment (4 or 6 days) with increasing aurantioobtusin concentrations led to a slight suppression of viral replication, this effect was consistently accompanied by observable cytotoxicity, which complicates the interpretation of its specific antiviral activity.

**Fig 3:**
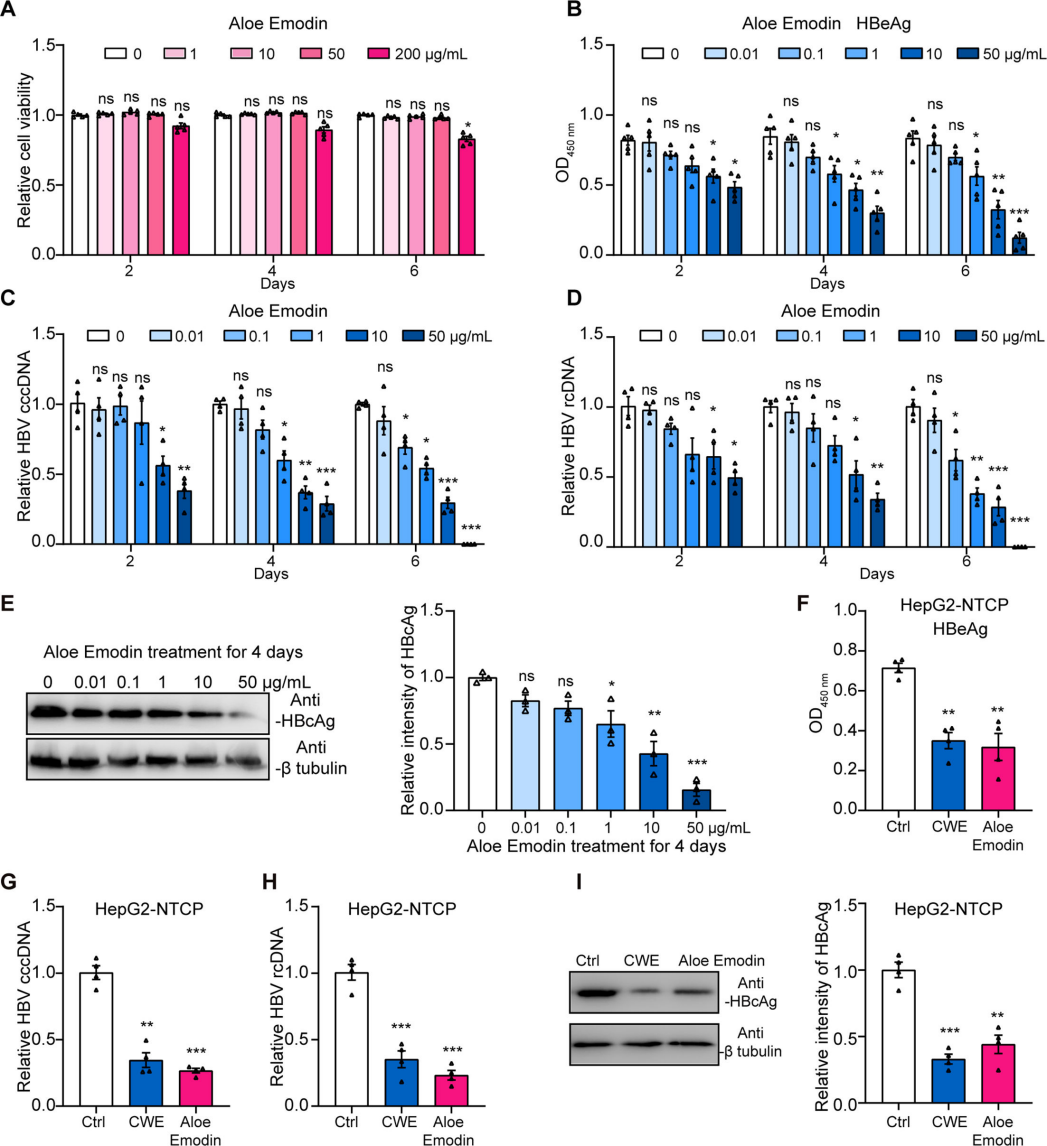
Aloe emodin treatment suppresses HBV replication. (**A**) Cell proliferation of HepAD38 cells treated with different doses of aloe emodin (0, 1, 10, 50, and 200 μg/mL) for 2, 4, and 6 days was determined by CCK-8 assay. (**B**) ELISA analysis of HBeAg amounts in the culture medium of HepAD38 cells treated with aloe emodin (0, 0.01, 0.1, 1, 10, and 50 μg/mL) for 2, 4, and 6 days. (**C and D**) The levels of HBV cccDNA (**C**) and rcDNA (**D**) in HepAD38 cells treated with aloe emodin (0, 0.01, 0.1, 1, 10, and 50 μg/mL) for 2, 4, and 6 days. (**E**) Whole cell lysates from HepAD38 cells treated with aloe emodin (0, 0.01, 0.1, 1, 10, and 50 μg/mL) for 4 days were subjected to Western blotting analysis to detect HBcAg amounts. (**F**) ELISA analysis of HBeAg amounts in the culture medium of HepG2-NTCP cells treated with 50 μg/mL CWE or 10 μg/mL aloe emodin for 4 days. (**G and H**) The levels of HBV cccDNA (**G**) and rcDNA (**H**) in HepG2-NTCP cells treated with 50 μg/mL CWE or 10 μg/mL aloe emodin for 4 days. (**I**) Whole cell lysates from HepG2-NTCP cells treated with 50 μg/mL CWE or 10 μg/mL aloe emodin for 4 days were subjected to Western blotting analysis to detect HBcAg amounts. The relative intensities of HBcAg bands were quantified using ImageJ software. Data are presented as mean ± SEM from three independent experiments. ns, no significant difference; *P < 0.05; **P < 0.01; ***P < 0.001 (one-way ANOVA).

In marked contrast, another anthraquinone compound, aloe emodin, demonstrated a favorable safety profile coupled with potent antiviral activity. Even at the highest tested concentration (200 μg/mL), aloe emodin only slightly influenced HepAD38 cell proliferation over the 6-day period (Fig. 3A). Strikingly, virological assessment revealed that aloe emodin treatment produced rapid and robust suppression of HBV replication. Significant reductions in HBeAg secretion (Fig. 3B), as well as intracellular cccDNA and rcDNA levels (Fig. 3C and D), were observed even after short-term exposure. This antiviral effect was further enhanced with increasing concentrations and prolonged treatment durations, demonstrating clear dose and time dependence. Western blotting analysis further corroborated these findings, showing a significant decrease in intracellular HBcAg amounts following 4 days of aloe emodin treatment (Fig. 3E). Collectively, these findings revealed clear distinctions among the tested compounds. While cassiaside C, rubrofusarin-6-O-β-gentiobioside, and aurantioobtusin exhibit only marginal anti-HBV activity that is either limited by cytotoxicity or requires prolonged exposure, aloe emodin emerges as a promising candidate with low cytotoxicity and potent, time-dependent antiviral efficacy against HBV replication.

To further validate the anti-HBV effects of CWE and aloe emodin, we employed the HepG2-NTCP cell model, which differs fundamentally from the HepAD38 system. HepAD38 cells contain a tetracycline-inducible integrated HBV genome and support only late replication steps (e.g., rcDNA synthesis and virion production) but lack the functional HBV receptor, thus preventing viral entry and cccDNA formation (23). In contrast, HepG2-NTCP cells overexpress the NTCP receptor, rendering them fully susceptible to HBV infection and enabling the entire viral life cycle, including entry, cccDNA establishment, and progeny release (24, 25). Following HBV infection, HepG2-NTCP cells were treated with CWE or aloe emodin for 4 days. As shown in Fig. 3F through I, both compounds significantly reduced extracellular HBeAg levels as well as intracellular cccDNA, rcDNA, and HBcAg. These results are consistent with those observed in HepAD38 cells (23), confirming that the antiviral activity of CWE and aloe emodin is reproducible in a physiologically relevant full-life-cycle model (24, 25).

### Exposure to CWE or aloe emodin leads to robust activation of the type I interferon signaling pathway

**Fig 4:**
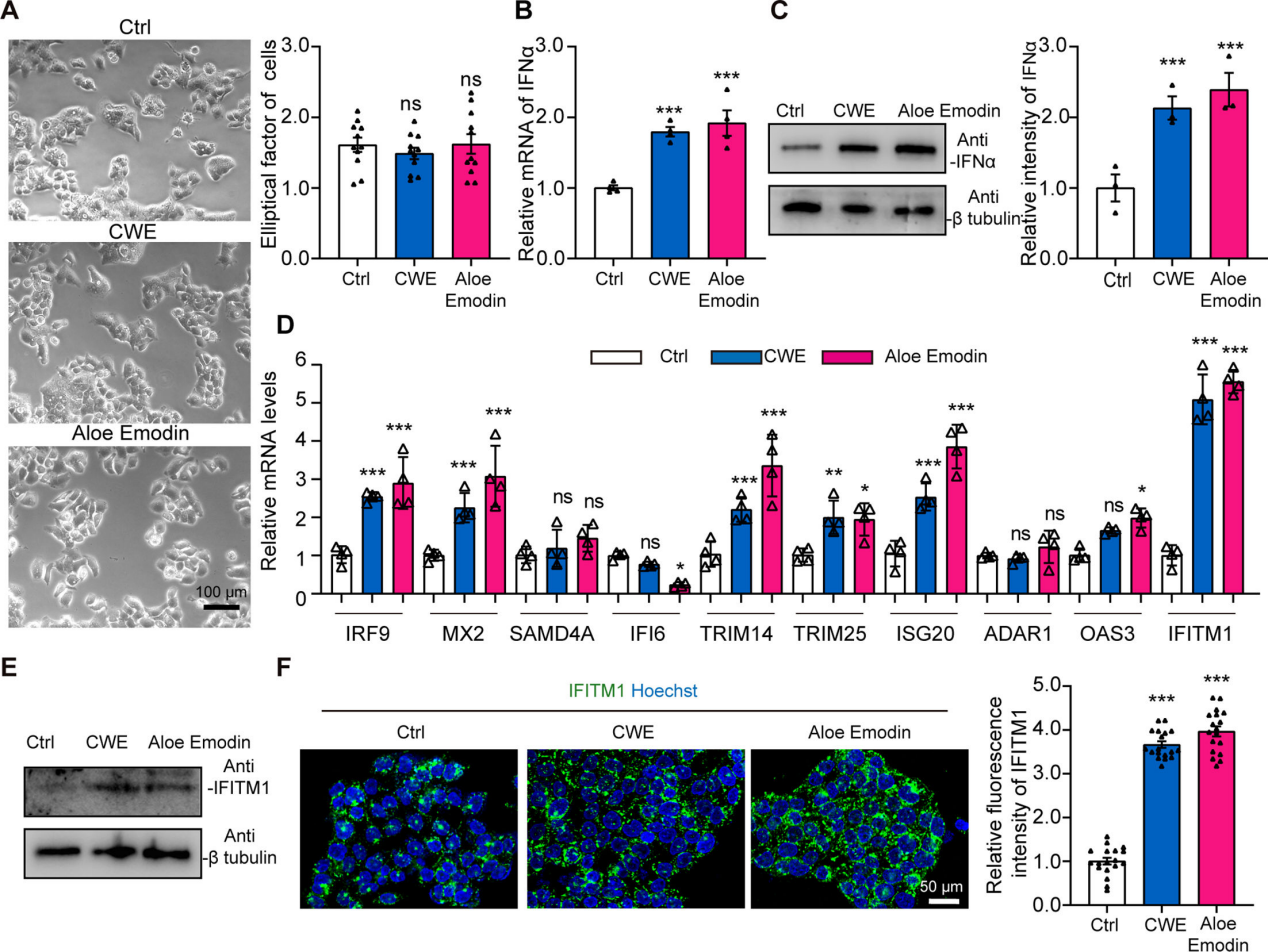
The type I interferon signaling pathway is markedly activated in response to CWE or aloe emodin treatment. (**A**) Neither CWE nor aloe emodin treatment alters the morphology of HepAD38 cells. Phase contrast microscopy images show the morphology of HepAD38 cells treated with 50 μg/mL CWE or 10 μg/mL aloe emodin for 4 days. Scale bar: 100 µm. The elliptical factor of cells is calculated as the ratio of the long axis to the short axis after measuring the length and width using ImageJ software. (**B**) Treatment with either CWE or aloe emodin induces the expression of IFNα. HepAD38 cells were treated with 50 μg/mL CWE or 10 μg/mL aloe emodin for 4 days, and the intracellular IFNα mRNA levels in each group were measured by qRT-PCR. (**C**) Western blotting analysis of endogenous IFNα in HepAD38 cells treated with 50 μg/mL CWE or 10 μg/mL aloe emodin for 4 days. The relative intensities of IFNα bands were quantified using ImageJ software. (**D**) A limited screen was performed to identify candidate ISGs with altered expression in HepAD38 cells treated with 50 μg/mL CWE or 10 μg/mL aloe emodin for 4 days. The intracellular ISGs mRNA amounts in each group were measured by qRT-PCR. (**E**) Treatment with either CWE or aloe emodin results in increased expression of IFITM1. Western blotting analysis of IFITM1 in HepAD38 cells treated with 50 μg/mL CWE or 10 μg/mL aloe emodin for 4 days. IFITM1 intensities in the indicated group were quantified by ImageJ. (**F**) HepAD38 cells, treated with 50 μg/mL CWE or 10 μg/mL aloe emodin for 4 days, were fixed, immunostained with IFITM1 antibody, and subjected to confocal microscopy. IFITM1 fluorescence intensities in the indicated group were quantified by ImageJ. *n* = 18 cells for each group. Scale bar, 50 µm. Data are presented as mean ± SEM from three independent experiments. ns, no significant difference; *P < 0.05; **P < 0.01; ***P < 0.001 (one-way ANOVA).

Based on the aforementioned findings that CWE and its bioactive anthraquinone extracts exhibit anti-HBV activity, we next sought to elucidate the molecular mechanisms underlying their antiviral effects. Type I interferon, particularly pegylated IFNα, serves as a well-established therapeutic modality for chronic hepatitis B (4, 5). To determine whether the antiviral activities of CWE and aloe emodin are associated with the modulation of the type I interferon pathway, we quantified intracellular IFNα expression in HepAD38 cells following 4 days of treatment with CWE (0 and 50 μg/mL) or aloe emodin (0 and 10 μg/mL). Prior to evaluating IFNα expression, we assessed the safety of CWE and aloe emodin by examining their effects on cell morphology and growth status. As shown in Fig. 4A, neither CWE at 50 μg/mL nor aloe emodin at 10 μg/mL affected cellular viability or morphology, confirming the safety of these concentrations for subsequent experiments. As illustrated in Fig. 4B and C, treatment with either CWE or aloe emodin significantly upregulated intracellular IFNα mRNA and protein levels compared to the untreated control group. What is more, we treated uninfected HepG2 cells with 50 μg/mL CWE for 4 days and measured the mRNA and protein levels of IFNα. As shown in Fig. S1A and B, both compounds induced a modest upregulation of IFNα in uninfected HepG2 cells. However, the magnitude of this upregulation was substantially lower than that observed in HBV-infected HepAD38 cells treated with the same compounds, suggesting that viral infection itself contributes significantly to the stronger immune responses. These results imply that the type I interferon pathway may be involved in mediating the anti-HBV effects of bioactive constituents derived from *Cassiae* semen.

To further delineate the downstream signaling events of the type I interferon pathway activated by CWE and aloe emodin, we examined interferon regulatory factor 9 (IRF9), a key transcription factor that drives the expression of ISGs via interferon-stimulated response element (ISRE) (26, 27), as well as a panel of type I interferon-stimulated genes with known anti-HBV activity, including interferon-induced GTP-binding protein (MX2) (28), protein Smaug homolog 1 (SAMD4A) (29), interferon alpha-inducible protein 6 (IFI6) (30), tripartite motif-containing proteins (TRIM14 and TRIM25) (31), interferon-stimulated gene 20 kDa protein (ISG20) (32), double-stranded RNA-specific adenosine deaminase (ADAR1) (33), 2′-5′-oligoadenylate synthase 3 (OAS3) (34), and IFITM1 (6, 35). We found that CWE or aloe emodin treatment markedly enhanced IRF9 transcription; among the screened ISGs, IFITM1 exhibited the most prominent upregulation (Fig. 4D). Given our previous studies demonstrating that IFITM1 functions as a potent restriction factor for HBV replication (6, 7), we hypothesized that IFITM1 might act as a downstream effector in the antiviral response induced by *Cassiae* semen-derived bioactive constituents.

To test this, we evaluated the expression of IFITM1 under identical treatment conditions. Immunoblotting and immunofluorescence staining analyses consistently revealed that both CWE and aloe emodin significantly upregulated IFITM1 protein expression (Fig. 4E and F). Then, we treated uninfected HepG2 cells with 10 μg/mL AE for 4 days and assessed the mRNA and protein levels of IFITM1. As shown in Fig. S1C and D, both compounds induced only a mild upregulation of IFITM1 in uninfected HepG2 cells. This result is consistent with the trend of IFNα in HepG2 cells, collectively indicating that the presence of viral infection itself contributes markedly to the stronger immune responses. Collectively, these findings indicate a model wherein CWE and aloe emodin may suppress HBV replication at least in part through an IFITM1-dependent mechanism, which is potentially initiated by the activation of the type I interferon signaling pathway.

### The inhibitory effect of CWE and aloe emodin on HBV replication is mediated by the induction of IFITM1

**Fig 5:**
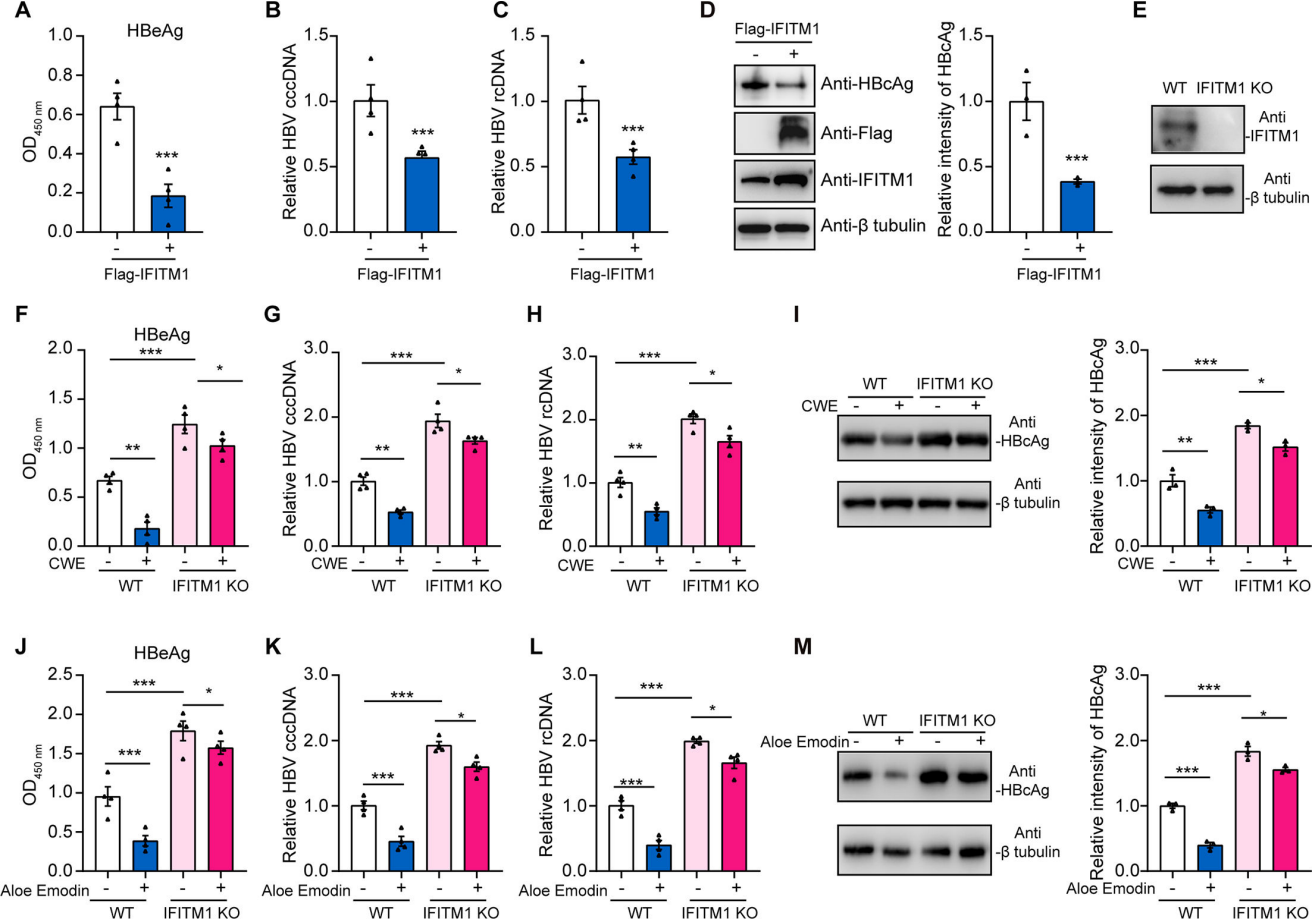
CWE or aloe emodin upregulates IFITM1 to suppress HBV replication. (**A**) Overexpression of Flag-IFITM1 significantly decreases the levels of HBeAg. Analysis of HBeAg amounts in the culture medium of HepAD38 cells was analyzed by ELISA. (**B and C**) Overexpression of Flag-IFITM1 significantly decreases the levels of HBV cccDNA (**B**) and rcDNA (**C**). The intracellular HBV cccDNA (**B**) and rcDNA (**C**) in Flag- or Flag-IFITM1-expressing HepAD38 cells were analyzed by qRT-PCR. (**D**) Overexpression of Flag-IFITM1 reduces HBcAg protein levels. Whole cell lysates from HepAD38 cells expressing Flag- or Flag-IFITM1 were subjected to western blotting analysis. (**E**) Validation of IFITM1 knockout efficiency in HepAD38 cells. Western blotting analysis was performed to assess the expression levels of endogenous IFITM1 protein in both wild-type (WT) and IFITM1 knockout (IFITM1 KO) HepAD38 cells. (**F**) ELISA analysis of HBeAg in WT and IFITM1 KO HepAD38 cells treated without and with 50 μg/mL CWE for 4 days. (**G and H**) Knockout of IFITM1 attenuates the inhibitory effect of CWE treatment on HBV replication. WT and IFITM1 KO HepAD38 cells were treated with or without 50 μg/mL CWE for 4 days, and the intracellular HBV cccDNA (**G**) and rcDNA (**H**) levels in each group were measured by qRT-PCR. (**I**) Western blotting analysis of HBcAg in WT and IFITM1 KO HepAD38 cells treated without and with 50 μg/mL CWE for 4 days. HBcAg intensities in the indicated group were quantified by ImageJ. (**J**) ELISA analysis of HBeAg in WT and IFITM1 KO HepAD38 cells treated without and with 10 μg/mL aloe emodin for 4 days. (**K and L**) IFITM1 knockout blunts aloe emodin-mediated inhibition of HBV replication. WT and IFITM1 KO HepAD38 cells were treated with or without 10 μg/mL aloe emodin for 4 days, and the intracellular HBV cccDNA (**K**) and rcDNA (**L**) amounts in each group were measured by qRT-PCR. (**M**) Western blotting analysis of HBcAg in WT and IFITM1 KO HepAD38 cells treated without and with 10 μg/mL aloe emodin for 4 days. The relative intensities of HBcAg bands were quantified using ImageJ software. Data are presented as mean ± SEM from three independent experiments. ns, no significant difference; *P < 0.05; **P < 0.01; ***P < 0.001 (unpaired two-tailed *t*-test for panels **A–C**; one-way ANOVA for panels** E–J**).

IFITM1 has emerged as a novel anti-HBV factor over the past two years. Both Ye et al. and our group have demonstrated that IFITM1 exerts inhibitory effects on HBV replication and infection in multiple cellular models, including HepG2.2.15 cells (stably harboring the integrated HBV genome) and HepG2-NTCP cells (supporting the complete HBV life cycle from viral entry to replication and secretion) (6, 35). However, the molecular and pharmacological regulatory mechanisms governing IFITM1 in this process remain largely elusive. Herein, we overexpressed IFITM1 in HepAD38 cells to investigate its effect on HBV replication. The results showed that IFITM1 overexpression significantly reduced extracellular HBeAg levels as well as intracellular cccDNA, rcDNA, and HBcAg levels in HepAD38 cells, confirming the inhibitory role of IFITM1 in HBV replication (Fig. 5A through D). These findings are consistent with our previous observations in HepG2.2.15 cells (6).

To verify whether the bioactive constituents of *Cassiae* semen exert anti-HBV replication activity via IFITM1, we constructed an IFITM1 knockout (KO) HepAD38 cell line using CRISPR-Cas9 technology, with knockout efficiency confirmed by western blotting analysis (Fig. 5E). Subsequently, we performed a series of experiments where both wild-type (WT) and IFITM1 KO HepAD38 cells were incubated with or without 50 μg/mL CWE for 4 days. Following this treatment period, extracellular (HBeAg) and intracellular (cccDNA, rcDNA, and HBcAg) levels of key HBV replication markers were quantitatively assessed (Fig. 5F through I). Compared with WT cells, IFITM1 KO cells exhibited significantly elevated levels of HBV HBeAg, cccDNA, rcDNA, and HBcAg, highlighting the pivotal role of IFITM1 in suppressing HBV replication. Importantly, 50 μg/mL CWE, which effectively inhibited viral replication in WT cells, exerted only a modest inhibitory effect on these viral markers in IFITM1 KO cells, which indicates that the antiviral activity of CWE is functionally dependent on IFITM1.

Using an identical experimental setup, we further evaluated the effect of 10 μg/mL aloe emodin on IFITM1 KO HepAD38 cells. Consistent with the results from CWE treatment, the absence of IFITM1 attenuated the anti-HBV activity of aloe emodin (Fig. 5J through M). Notably, a slight reduction in HBV HBeAg, cccDNA, rcDNA, and HBcAg levels was still observed in IFITM1 KO cells treated with either CWE or aloe emodin, suggesting that additional immune-related factors may be involved in the anti-HBV effects of CWE and aloe emodin. Thus, we treated IFITM1 knockout HepAD38 cells with varying concentrations of CWE (0, 0.1, 1, 10, 50, and 100 μg/mL) and aloe emodin (0, 0.01, 0.1, 1, 10, and 50 μg/mL) for 4 days. Intracellular levels of cccDNA and rcDNA were then measured. The results showed that only relatively high concentrations—50 and 100 μg/mL of CWE, and 10 and 50 μg/mL of aloe emodin—exerted modest inhibitory effects on HBV replication (Fig. S2A through D), which implies that other factors are involved. Collectively, these findings provide compelling evidence that both CWE and aloe emodin inhibit HBV replication by upregulating IFITM1.

## DISCUSSION

**Fig 6:**
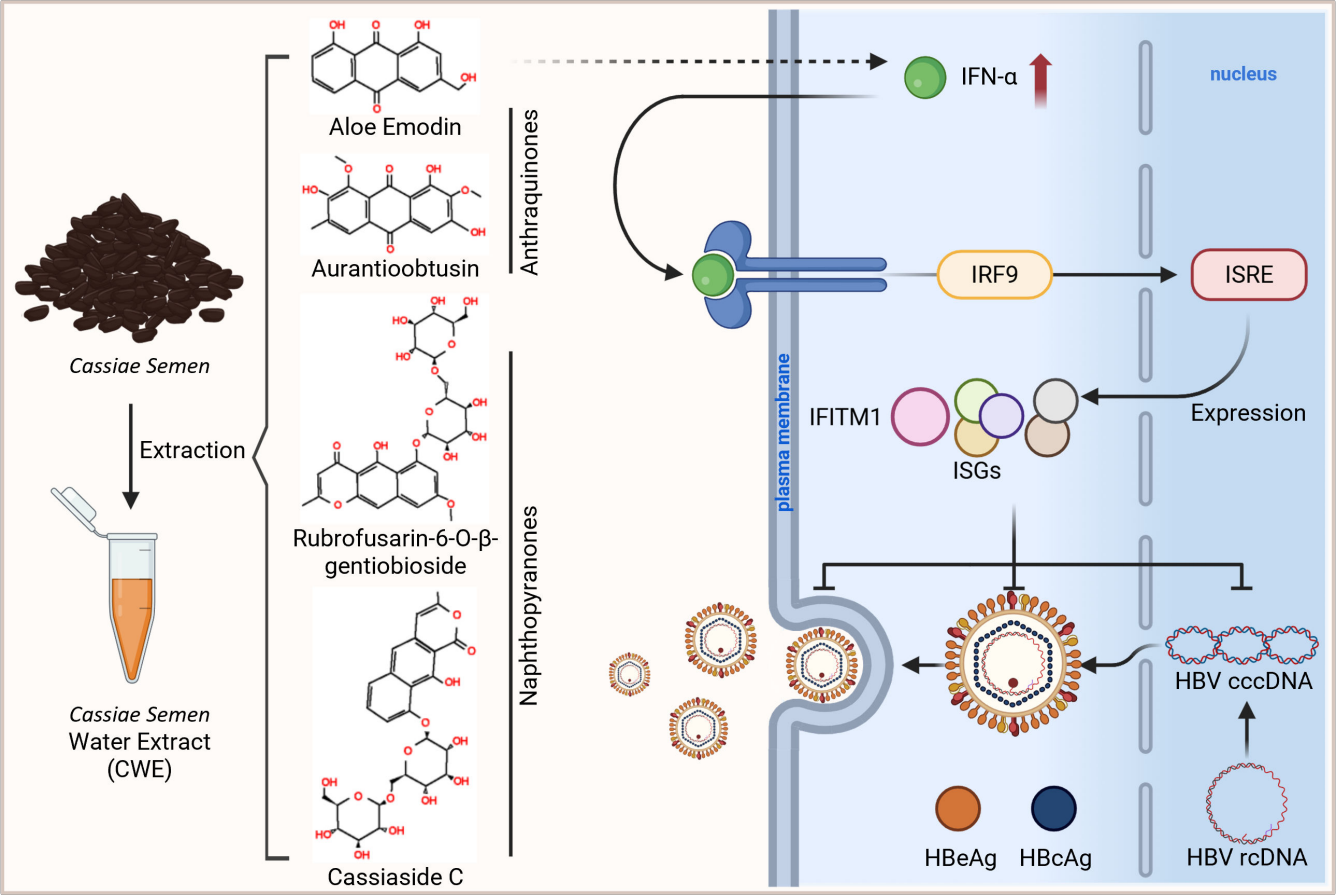
The bioactive compound aloe emodin derived from *Cassiae* semen exerts the inhibitory effect on HBV replication through upregulation of the IFN-IFITM1 signaling pathway.

Our study demonstrates that both *Cassiae* semen water extract and its purified bioactive component aloe emodin significantly inhibit HBV replication *in vitro*. The antiviral mechanism is primarily mediated through the activation of the IFNα signaling cascade, which subsequently upregulates the expression of the IFN-stimulated restriction factor IFITM1 (Fig. 6). These findings not only elucidate a novel anti-HBV molecular mechanism for a traditional herbal medicine but also establish a solid theoretical foundation for the development of *Cassiae* semen-based phytopharmaceuticals against chronic hepatitis B (CHB).

The antiviral function of *Cassiae* semen exists within a broader context of its well-documented pharmacological profiles. Beyond this newly identified anti-HBV action, *Cassiae* semen and its diverse array of bioactive constituents, including anthraquinones and naphthopyranones, are renowned for their hepatoprotective and potent antioxidant properties (8, 36). Fatty liver disease has emerged as a highly prevalent comorbidity in patients with CHB (37), which highlights the critical need for therapeutic strategies targeting concurrent liver lipid dysregulation in CHB management. Aligning with this clinical relevance, *Cassiae* semen extracts have been shown to exert a beneficial effect on fatty liver disease by inhibiting fatty acid synthesis and upregulating the autophagy signaling pathway (9). Besides, research indicates that the naphthopyrone compound derived from *Cassiae* semen*,* like cassiaside C (toralactone-9-O-β-D-gentiobioside), confers liver protection specifically through the activation of the Nrf2 (nuclear factor erythroid 2-related factor 2) antioxidative signaling pathway, a master regulator of cellular defense against oxidative stress (38). Given that fatty liver disease and oxidative stress are key drivers of CHB progression to cirrhosis and hepatocellular carcinoma (HCC) (39), the integrated pharmacodynamic profiles of *Cassiae* semen, combining anti-HBV, anti-steatotic, and antioxidant effects, position it as a unique therapeutic candidate for CHB treatment, which can simultaneously address multiple pathological links of the disease.

While the CWE exhibited the most favorable cytotoxicity profile, its highly complex chemical composition presents challenges for standardization and mechanistic precision. The current comparative analysis of the isolated bioactive constituents underscores that aloe emodin stands out as the most promising lead compound for anti-HBV drug development. Aloe emodin demonstrated antiviral potency and a rapid onset of action, effectively suppressing HBV replication within 2 days at a concentration of 10 μg/mL and nearly abolishing infection at 50 μg/mL over 6 days, with only minimal associated cytotoxicity at these therapeutically effective doses. In contrast, compounds such as cassiaside C, aurantioobtusin, and rubrofusarin-6-O-β-gentiobioside displayed either delayed antiviral effects (appearing only after 4–6 days) or weaker activity, and critically, they manifested significant cytotoxicity within the very concentration and time ranges necessary for their antiviral action, thus diminishing their therapeutic window and clinical potential. Although aloe emodin exhibits slightly higher cytotoxicity than CWE (likely due to the loss of synergistic buffering effects in the natural matrix), it shows a more favorable safety profile compared to other purified compounds (e.g., cassiaside C, aurantioobtusin) at therapeutically effective doses, rendering it an excellent candidate for further structural optimization. The suitability of aloe emodin is further supported by emerging evidence on the broad-spectrum anti-pathogen capabilities of *Cassiae* semen components, including reported inhibition of SARS-CoV-2 infection and antibacterial actions against drug-resistant strains (8, 17).

While the present study is the first to identify aloe emodin from *Cassia* seed extract as possessing anti-HBV activity, it is noteworthy that Parvez et al. systematically evaluated the anti-HBV potential of *Aloe vera* and its anthraquinone extracts in 2019, with their work confirming aloe emodin to be the most promising natural anti-HBV constituent (40). Our results demonstrate that aloe emodin isolated from *Cassiae* semen exerts anti-HBV effects by activating the type I IFN signaling pathway to upregulate IFITM1 expression. In contrast, Parvez et al. reported that aloe emodin from *Aloe vera* directly interacts with HBV polymerase to inhibit viral replication, accompanied by enhancing efficacy when combined with lamivudine. These discrepancies highlight the source-dependent pharmacological characteristics of aloe emodin and its dual potential as an immunomodulator or direct viral enzyme inhibitor, providing complementary insights for developing aloe emodin-based anti-HBV therapeutics with distinct mechanisms of action.

Placing the present findings within the wider landscape of TCM research against HBV reveals both convergent strategies and distinct mechanistic identities. The immunomodulatory mechanism elucidated for *Cassiae* semen, activating the host’s innate IFN response, resonates with the mode of action of other TCM-derived agents. For instance, saponins isolated from *Abrus cantoniensis* Hance have been shown to reduce HBV DNA and antigen levels partly by modulating host metabolic pathways and enhancing immune responses, including the production of type II IFN (41). However, we have previously reported that other prominent TCM components operate through entirely different pathways. A prime example is epigallocatechin-3-gallate (EGCG) from green tea, which functions as an antagonist of the host nuclear receptor FXRα (farnesoid X receptor alpha), thereby suppressing the transcriptional activity of HBV promoter (19, 42). This diversity highlights the promising anti-HBV strategies within the TCM pharmacopeia.

Modern network pharmacology analyses consistently illustrate that the therapeutic efficacy of complex TCM or common flavonoid constituents (e.g., quercetin and luteolin) against HBV and HBV-related HCC arises from characteristically multi-target, multi-pathway interactions (20, 43). While this polypharmacology offers the advantage of broad-spectrum activity and potential reduction of drug resistance, it may also increase the risk of unpredictable off-target effects. In the current research, the mechanism identified for CWE, and particularly for aloe emodin, represents a somewhat more targeted approach. By upregulating the type I IFN pathway, the aqueous extract of *Cassiae* semen and its key bioactive component aloe emodin induce the expression of several downstream ISGs, among which the most prominently upregulated one is IFITM1, a well-characterized broad-spectrum antiviral protein. Future studies could further explore whether treatment with CWE or aloe emodin modulates the infection of other viruses through ISGs, including IFITM1.

Over the past decade, a growing number of interferon-stimulated genes have been reported to be involved in regulating the replication and infection of HBV (4, 5). In the present study, we performed a small-scale screening and found that IFITM1 exhibited the most pronounced response to treatments with CWE and aloe emodin. Additionally, we revealed that the bioactive components of *Cassiae* semen exert an anti-HBV replication effect by upregulating IFITM1 expression. Notably, we found that ISGs such as MX2, TRIM14, and ISG20 were also upregulated following treatment with CWE and aloe emodin. The reduction of IFI6 mRNA by aloe emodin appears counterintuitive, given the known antiviral function of IFI6 (30). One possible explanation is that aloe emodin exerts its antiviral activity primarily through other interferon-stimulated genes (e.g., IFITM1) rather than IFI6. Additionally, ISG regulation is highly context-dependent; aloe emodin may activate a distinct subset of the IFN pathway or trigger negative feedback mechanisms that selectively suppress IFI6 while inducing other antiviral effectors. Further studies are needed to clarify this regulatory divergence. Furthermore, knockout of IFITM1 did not completely abrogate the anti-HBV activity of *Cassiae* semen components, indicating that other ISGs may also be involved in mediating the antiviral effects of *Cassiae* semen extracts. In-depth identification and characterization of the targeted factors of *Cassiae* semen extract in the innate immune and interferon signaling pathways will provide novel insights into the prevention and treatment of viral diseases.

Previously, Ye et al. and we have reported that the anti-HBV replication activity of IFITM1 is regulated by the JAK-STAT signaling pathway and the level of its intrinsic S-palmitoylation post-translational modification (6, 35), while IFITM3 promotes HBV entry through interacting with the HBV receptor Na^+^/taurocholate co-transporting polypeptide (NTCP) (44). The broad-spectrum antiviral IFITM members exert their antiviral activity primarily through multiple mechanisms: modulating the physical properties of the cell plasma membrane to inhibit virus-host membrane fusion, thereby preventing the viral genome from entering the cytoplasm; downregulating the transcription and translation of viral proteins; and directly binding to viral structural proteins to interfere with viral infection (45). However, the specific molecular mechanisms underlying the interaction between IFITM1 and HBV genes or proteins remain unelucidated. Subsequent studies should focus on filling this research gap, developing targeted drugs (enhancing IFITM1 activity or blocking IFITM3-NTCP binding), and exploring their potential as therapy-responsive biomarkers.

In conclusion, this study demonstrates for the first time that *Cassiae* semen water extract and its key bioactive component aloe emodin potently inhibit HBV replication in HepAD38 cells via activating the type I interferon pathway and upregulating the antiviral factor IFITM1. These findings elucidate a novel anti-HBV mechanism of *Cassiae* semen and offer a valuable paradigm that bridges traditional herbal medicine with a modern, mechanism-driven drug discovery strategy for combating persistent HBV infections.

## MATERIALS AND METHODS

### Cell culture and transfection

HepG2, HepAD38, and HepG2-NTCP cells were all maintained in high-glucose Dulbecco’s modified Eagle’s medium (DMEM; #11965092, Gibco, USA) supplemented with 10% fetal bovine serum (FBS), 10 μg/mL streptomycin, 10 U/mL penicillin, and 4 mM L-glutamine at 37°C in a humidified atmosphere containing 5% CO₂. HepAD38 cells were a gift from Professor Yuchen Xia (Wuhan University, Wuhan, China). To trigger hepatitis B virus (HBV) production, HepAD38 cells were cultured in the aforementioned medium without 1 µg/mL doxycycline (#HY-N0565, MCE, USA). Additionally, HepG2-NTCP cells were cultured in the same basic medium with the addition of 8 μg/mL blasticidin S (#S46315, Yuanye Bio-Technology, China). For cell passaging, cells were rinsed with pre-warmed PBS, detached with 0.25% trypsin and 0.02% EDTA for 3 min, neutralized with complete medium, and centrifuged at 800 *× g* for 5 min.

For transfection, 3 × 10⁵ cells were seeded in six-well plates. After 24 h, 2.5  µg plasmid DNA was transfected using jetPRIME transfection reagent (#101000015; Polyplus, France) at a reagent-to-DNA ratio of 3:1. Transfection efficiency was evaluated 24 h post-transfection by fluorescence microscopy or western blotting.

### Aqueous extraction of *Cassiae* semen

The *Cassiae* semen water extract (CWE) was prepared using the method described by Kim et al. (14) and modified by our laboratory as follows: 200 g of dried *Cassiae* semen seeds were immersed in eight volumes of distilled water at 95°C for 2 h. The resulting supernatant was filtered through five layers of sterile gauze, centrifuged at 10,000 *× g* for 15 min, and further filtered through a 0.22 μm sterile filter. The filtrate was frozen at −80°C for more than 48 h and subsequently lyophilized for over 24 h to achieve complete dryness. The lyophilized CWE was weighed accurately and stored at −20°C in the dark until use.

### Western blotting

Transfected cells were lysed in RIPA buffer (#P0013, Beyotime, China) containing 20 mM Tris (pH 7.5), 150 mM NaCl, 1% Triton X-100, EDTA, Na_3_VO_4_, leupeptin, and Protease inhibitor cocktail (#P1008, Beyotime). Cell lysates were clarified by centrifugation at 10,000 *× g* for 15 min at 4°C, denatured with Laemmli sample buffer at 95°C for 5 min, separated by 12.5% SDS-PAGE, and transferred onto 0.45 µm PVDF membranes (#IPVH00010, Millipore, Germany). After blocking with 5% BSA for 1 h at room temperature, membranes were probed with antibodies against β-tubulin (dilution: 1:5,000; #66240-1, Proteintech, USA), IFN Alpha (dilution: 1:3,000; #66162-1, Proteintech), IFITM1 (dilution: 1:3,000; #ab233545, Abcam, USA), and HBcAg (dilution: 1:3,000; #ab316283, Abcam). HRP-conjugated secondary antibodies were used for detection with the West Femto ECL kit (#PE0030, Solarbio, China), and band intensities were quantified using ImageJ 1.54 r (National Institutes of Health, USA).

### Enzyme-linked immunosorbent assay (ELISA) for HBeAg

HepAD38 cells were treated with CWE, cassiaside C, aurantioobtusin, aloe emodin, and rubrofusarin-6-O-β-gentiobioside at various concentrations for 2, 4, and 6 days. Cell culture medium was collected, and the level of HBeAg was measured using ELISA kits (#MM-51632H2, MeiMian, China) according to the manufacturer’s instructions. Absorbance was read at 450 nm using a microplate reader, and relative HBeAg levels were calculated based on the absorbance values.

### Extraction and quantification of HBV DNA forms

The genomic DNA from HepAD38 cells, cultured for 2, 4, and 6 days, was extracted using the genomic DNA purification kit (#2868751, Thermo Scientific, USA). Quantifying HBV cccDNA is technically challenging due to its extremely low abundance (~1–10 copies/cell), identical sequence to other HBV DNA forms, and high thermal stability, which complicates PCR-based detection (46). To ensure specificity, total genomic DNA was digested with Mung Bean Nuclease (#M0250S, NEB, USA) to eliminate single-stranded DNA (ssDNA). Subsequently, cccDNA was quantified by qRT-PCR using primers spanning the rcDNA gap region, enabling selective amplification of fully double-stranded cccDNA over other HBV DNA species.

### Quantitative real-time PCR (qRT-PCR)

Total RNA from cells treated with compounds for 2, 4, and 6 days was extracted with Super FastPure Cell RNA Isolation Kit (#RC102-01, Vazyme, China), reverse-transcribed using HisyGo RT Red SuperMix for qPCR (+gDNA Wiper) (#RT101-01, Vazyme) to eliminate genomic DNA contamination, and amplified with HQ SYBR qPCR mix (#ZF503-2, ZomanBio, China) on the StepOne Plus Real-Time PCR System (Applied Biosystem, USA). Gene expression was analyzed via the 2^−ΔΔCt^ method and normalized to the level of GAPDH. Each experimental transcript was checked in quintuplicate. Samples without cDNA template served as negative controls. All primers are listed in Table S1.

### Cell viability assay

The cytotoxicity of CWE, cassiaside C, aurantioobtusin, aloe emodin, and rubrofusarin-6-O-β-gentiobioside was evaluated with an enhanced CCK-8 assay (#MC0301-1000, Kermey, China). Briefly, 2 × 10⁴ HepAD38 cells were seeded into 96-well plates and pre-cultured for 24 h at 37 °C under 5% CO₂. Following the addition of test compounds at varying concentrations, the plates were further incubated for 2, 4, or 6 days. The culture medium containing the compounds was changed every other day. On the last day, 10 μL of CCK-8 solution was carefully added to each well and incubated for 2 h at 37 °C. Absorbance at 450 nm was measured using a microplate reader. For delayed measurement, 10 μL of 0.1 M HCl or 1% (wt/vol) SDS was added to each well to terminate the reaction, and plates were stored at room temperature in the dark; absorbance values remained stable within 24 h. Each experiment was performed in triplicate, and cell viability was expressed as a percentage relative to the untreated control group.

### Bioactive constituents treatment

HepAD38 cells were treated with cassiaside C (#119170-52-4, Hengchengzhiyuan, China), aurantioobtusin (#67979-25-3, Hengchengzhiyuan), aloe emodin (#481-72-1, Hengchengzhiyuan), and rubrofusarin-6-O-β-gentiobioside (#24577-90-0, Hengchengzhiyuan) at gradient concentrations for 2, 4, and 6 days in both cell viability and HBV-related assays. In the CCK-8 assay, the concentrations of CWE were 0, 10, 100, 1,000, and 10,000 μg/mL, while the concentrations of cassiaside C, rubrofusarin-6-O-β-gentiobioside, aurantioobtusin, and aloe emodin were 0, 1, 10, 50, and 200 μg/mL. In the anti-HBV drug concentration screening assay, the concentrations of CWE were 0, 0.1, 1, 10, 50, and 100 μg/mL; the concentrations of cassiaside C, rubrofusarin-6-O-β-gentiobioside, and aloe emodin were 0, 0.01, 0.1, 1, 10, and 50 μg/mL; and the concentrations of aurantioobtusin were 0, 0.01, 0.1, 1, 5, and 10 μg/mL.

### Infection assay

For HBV virus concentration, the culture supernatant of HepAD38 cells was collected and centrifuged to remove cell debris. The supernatant was collected, and 10% PEG 8000 and 4 M NaCl were added. The mixture was incubated overnight on a shaker at 4°C, then centrifuged at 1,000 × *g* and 4°C for 30 min. The supernatant was discarded, and the precipitate was resuspended in serum-free DMEM, aliquoted, and stored at −80°C. For the infection procedure, HepG2-NTCP cells were seeded into cell plates and cultured for 24 h until adherence. According to the multiplicity of infection (MOI) of 0.1−1, the concentrated HBV virus solution was mixed with medium containing 4% PEG 8000, added to the cell wells, and incubated at 37°C for 24 h. The viral infection solution was discarded, the cells were washed three times with PBS, and fresh complete medium was replaced. After continuous culture for 3~7 days, HBeAg, HBcAg, HBV cccDNA, and HBV rcDNA could be detected to evaluate the infection efficiency.

### Immunofluorescence (IF)

HepAD38 cells were plated on 35 mm glass-bottom dishes precoated with 10 µg/mL fibronectin (#F2006, Merck, USA). Following fixation in 3.7% paraformaldehyde (PFA) for 20 min, the cells were simultaneously blocked and permeabilized using a solution containing 5% BSA and 0.2% Triton X-100. Immunostaining was performed by incubating with a rabbit anti-IFITM1 primary antibody (dilution: 1:200; #ab233545, Abcam), followed by a CoraLite-488-conjugated goat anti-rabbit secondary antibody. Nuclei were counterstained with Hoechst 33342 (#C1025; Beyotime) prior to mounting in an antifade medium (#P0126; Beyotime). Fluorescence images were captured on a Leica TCS SP8 confocal microscope with a 60*×* oil immersion objective.

### Generation of IFITM1 knockout HepAD38 cells

CRISPR/Cas9 was used to knock out IFITM1 in our previously published work (6). sgRNA targeting IFITM1 was designed using the CRISPR-ERA tool (47) and cloned into the LentiCRISPRV2 vector via BsmBI restriction enzyme sites. HepAD38 cells transfected with the recombinant vector were selected using 2 µg/mL puromycin dihydrochloride (#HY-B1743A, MCE). Monoclonal cells were obtained by the limited dilution culture method, and the knockout efficiency was confirmed by western blotting. The sequences of the sgRNA targeting IFITM1 were as follows: forward: 5′-CACCGTGATCACGGTGGACCTTGGA-3′; reverse: 5′-AAACTCCAAGGTCCACCGTGATCAC-3′.

### Statistical analysis

Data are expressed as mean ± SEM. Statistical comparisons between two groups were performed using an unpaired two-tailed *t*-test; multiple groups were compared via one-way ANOVA with Tukey’s *post hoc* test. Statistical significance was defined as *P < 0.05, **P < 0.01, ***P < 0.001; ns: not significant. Analyses were performed using GraphPad Prism 9.5 (GraphPad Software, USA). All experiments were independently repeated at least three times. Figure 6 was created with BioRender.com.

## Data Availability

All data relevant to the study are included in the article. Additional supporting data are available from the corresponding authors upon request.

## SUPPLEMENTAL MATERIAL

Supplemental material msphere.00312-26-s0001.docx Table S1; Figures S1 and S2.

ASM does not own the copyrights to Supplemental Material that may be linked to, or accessed through, an article. The authors have granted ASM a non-exclusive, world-wide license to publish the Supplemental Material files. Please contact the corresponding author directly for reuse.
