# Supplementary material for: Aloe emodin isolated from Cassiae semen upregulates IFITM1 to suppress hepatitis B virus replication via the interferon signaling pathway
Source: mSphere. 2026 Jun 11;11(6):e00312-26. doi: 10.1128/msphere.00312-26 (PMC13317195; doi:10.1128/msphere.00312-26)
Supplement: Supplemental material — Table S1; Figures S1 and S2. [file msphere.00312-26-s0001.docx]

**Supplementary Material**

**Table S1. The primers of qRT-PCR used in this study**

| Gene type | Gene name | Forward primer 5′ to 3′ | Reverse primer 5′ to 3′ |
| --- | --- | --- | --- |
| Endogenous Gene | IFNα | GGAGTTTGATGGCAACCAG | GAGTTCGGTGCAGAATTTGTC |
| Endogenous Gene | IRF9 | GCCCTACAAGGTGTATCAGTTG | TGCTGTCGCTTTGATGGTACT |
| Endogenous Gene | MX2 | AAGCAGTATCGAGGCAAGGA | TCGTGCTCTGAACAGTTTGG |
| Endogenous Gene | SAMD4A | ACACTTCCCCACAGAACATG | TCATACTGAGGCACAACGAC |
| Endogenous Gene | IFI6 | AAGGCGGTATCGCTTTTCTT | ATCGCAGACCAGCTCATCA |
| Endogenous Gene | TRIM14 | TACATTACAGACGCCATTGGAC | GGGCTGGTTTTCAACAAGGT |
| Endogenous Gene | TRIM25 | AGGGATGAGTTCGAGTTTCTGG | GTTTTTGAGGTCTATGGTGCTCT |
| Endogenous Gene | ISG20 | CTCCTGAAAGGCAAGCTGGT | CAGGCTGTTCTGGATGCTCT |
| Endogenous Gene | ADAR1 | GTCGTCAGCTTGGGAACA | CGCAGTCTGGGAGTTGTA |
| Endogenous Gene | OAS3 | CCGAACTGTCCTGGGCCTGATCC | CCCATTCCCCAGGTCCCATGTGG |
| Endogenous Gene | IFITM1 | ATCCTGTTACTGGTATTCGG | TATAAACTGCTGTATCTAGG |
| Viral Gene | HBV rcDNA | GGAGGGATACATAGAGGTTCCTTGA | GTTGCCCGTTTGTCCTCTAATTC |
| Viral Gene | HBV cccDNA | CCCCGTCTGTGCCTTCTC | CAGCTTGGAGGCTTGAACAGT |
| Reference Gene | GAPDH | GACCACAGTCCATGCCATCAC | GCCTGCTTCACCACCTTCTT |

**Supplementary Figure legends**


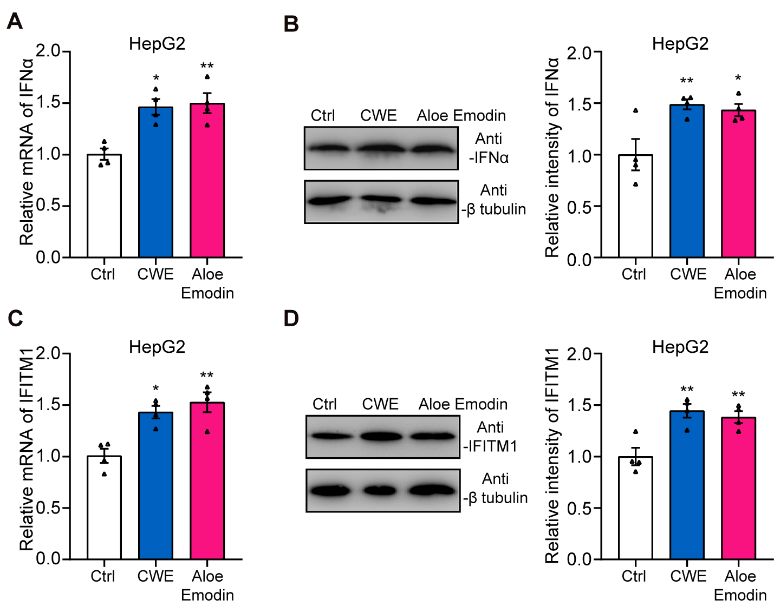


**Supplementary Fig 1** **Effects of CWE and Aloe Emodin treatment on IFNα and IFITM1 in HepG2 cells.** (A) Treatment with either CWE or Aloe Emodin slightly increases the expression of IFNα. HepG2 cells were treated with 50 μg/mL CWE or 10 μg/mL Aloe Emodin for 4 days, and the intracellular IFNα mRNA levels in each group were measured by qRT-PCR. (B) Western blotting analysis of endogenous IFNα in HepG2 cells treated with 50 μg/mL CWE or 10 μg/mL Aloe Emodin for 4 days. (C) Treatment with either CWE or Aloe Emodin increases the expression of IFITM1. HepG2 cells were treated with 50 μg/mL CWE or 10 μg/mL Aloe Emodin for 4 days, and the intracellular IFITM1 mRNA levels in each group were measured by qRT-PCR. (D) Western blotting analysis of endogenous IFITM1 in HepG2 cells treated with 50 μg/mL CWE or 10 μg/mL Aloe Emodin for 4 days. The relative intensities of IFNα bands were quantified using ImageJ software. Data are presented as mean ± SEM from three independent experiments. ns, no significant difference; **P* < 0.05; ***P* < 0.01 (one-way ANOVA).


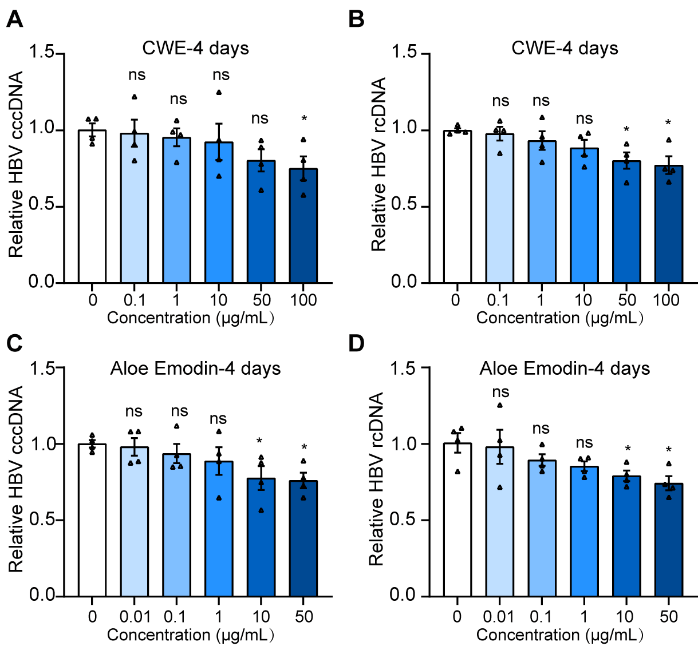


**Supplementary Fig 2** **The anti-HBV effects of CWE and Aloe Emodin treatment in IFITM1 KO HepAD38 cells.** (A and B) The levels of HBV cccDNA (A) and rcDNA (B) in IFITM1 KO HepAD38 cells treated with CWE (0, 0.1, 1, 10, 50, and 100 μg/mL) for 4 days. (C and D) The levels of HBV cccDNA (C) and rcDNA (D) in IFITM1 KO HepAD38 cells treated with Aloe Emodin (0, 0.01, 0.1, 1, 10, and 50 μg/mL) for 4 days. Data are presented as mean ± SEM from three independent experiments. ns, no significant difference; **P* < 0.05 (one-way ANOVA).
